# Intervention With WhatsApp Messaging to Compare the Effect of Self-Designed Messages and Standardized Messages in Adherence to Antiretroviral Treatment in Young People Living With HIV in a Hospital in Lima, Peru: Protocol for a Nonblinded Randomized Controlled Trial

**DOI:** 10.2196/66941

**Published:** 2025-05-22

**Authors:** Jeffrey Freidenson-Bejar, Dianne Espinoza, Rodrigo Calderon-Flores, Fernando Mejia, Elsa González-Lagos

**Affiliations:** 1 School of Medicine Universidad Peruana Cayetano Heredia Lima Peru; 2 Instituto de Medicina Tropical Alexander von Humboldt Universidad Peruana Cayetano Heredia Lima Peru; 3 Department of Infectious Diseases Hospital Nacional Cayetano Heredia Lima Peru

**Keywords:** mHealth, HIV, WhatsApp, adherence, ART, people living with HIV, young people, mobile phone messaging, digital app, messaging intervention, antiretroviral treatment, behavioral change technique, design, evaluation, self-design, randomized controlled trial, Peru

## Abstract

**Background:**

Young people living with HIV face challenges in consistently adhering to antiretroviral therapy (ART). Although mobile health interventions, particularly those using SMS text messaging, have been implemented to improve ART adherence, many lack a focus on specific behavioral mechanisms. Interventions incorporating behavioral change techniques (BCTs), especially those emphasizing customization, may enhance effectiveness. WhatsApp offers potential for delivering tailored, behaviorally grounded interventions with diverse communication features. We hypothesize that WhatsApp messages self-designed by participants, with spontaneous targeting of BCTs, could be more effective than standard WhatsApp messages designed by the researchers to improve ART adherence.

**Objective:**

The objective of this study is to evaluate the effectiveness of WhatsApp messages created by participants (self-designed) compared to WhatsApp messages designed by the researchers (standardized) over adherence to ART at 16 weeks of intervention in young people living with HIV who receive HIV care under routine conditions at a public hospital in Lima, Peru.

**Methods:**

A 2-arm randomized controlled trial with a parallel assignment of 1:1, with no blinding of study intervention, was performed. Eligible participants are consenting people living with HIV aged 18-29 years who receive HIV care at the study center and whose mobile phones support WhatsApp. Following informed consent and a baseline survey on clinical and personal preferences (eg, timing and frequency of messages), participants are randomized to the control group (messages designed by the research team) or to the experimental group (messages designed by participants), stratified by sex, educational level, current ART intake, and history of ART abandonment. Participants in both groups receive up to 3 WhatsApp messages per week for 16 weeks. ART adherence, the primary outcome, is measured using the Simplified Medication Adherence Questionnaire (SMAQ) at 4, 8, 12, and 16 weeks. Monthly feedback questionnaires on user experience are also administered. The WhatsApp chat format allows two-way communication between participants and the research team throughout the study. We will compare ART adherence between 2 groups at 16 weeks under the intention-to-treat principle, with no interim analysis planned. Based on an estimated 10% difference in adherence, 78.9% power, and a 2-sided α of .05, the target sample size was set at 120, later increased to 131 to include a 2-week pilot phase.

**Results:**

In March 2024, we started enrolling and randomizing participants. The study follow-up will continue until the last participant completes 16 weeks of intervention (November 2024). As of February 2025, we are in the process of data curation.

**Conclusions:**

This trial will compare the effectiveness of standardized vs self-designed WhatsApp messages on ART adherence measured at 16 weeks among young people living with HIV receiving routine care in a low-resource setting in Lima.

**Trial Registration:**

ClinicalTrials.gov NCT06500013; https://clinicaltrials.gov/ct2/show/NCT06500013

**International Registered Report Identifier (IRRID):**

DERR1-10.2196/66941

## Introduction

HIV programs continue in search of effective interventions to increase adherence to antiretroviral therapy (ART), especially for young people living with HIV/AIDS. This group faces increasing rates of new HIV infections [1] and heightened vulnerability to drivers of antiretroviral resistance, such as suboptimal ART adherence [2], ART abandonment, and non-retention in care [3].

Mobile health (mHealth) interventions tailored to young people living with HIV/AIDS seem highly promising, considering that so-called Gen Z tend to be digital natives who routinely use apps. At first glance, messaging interventions to improve adherence seem practical, low-cost, and prone to tailoring to users’ needs [4,5]. However, medication adherence involves complex behavioral aspects. Several behavioral change theories, notably the transtheoretical model, have been applied in interventions to improve medication adherence [6-8], often with contradictory findings [9,10]. The heterogeneity of findings may respond to messaging interventions operating simultaneously over different behavioral aspects, for instance, reminders and motivation. While a weekly frequency of delivery and a bidirectional platform have proven effective [11], the message’s content seems crucial to achieving behavioral changes [12].

Due to difficulties in identifying the specific factors responsible for behavioral changes, Abraham and Michie [13] developed a standardized system to isolate the drivers of behavior change: behavioral change techniques (BCTs). Since then, several interventions have adopted the BCT taxonomy to more accurately target specific behavioral aspects, improve the rigor and replication of behavioral studies, and enhance their reliability [14,15].

Most messaging interventions to improve adherence to ART have involved SMS and worked with standardized messages created by researchers. Currently, young people in low- and middle-income countries, including people living with HIV/AIDS, extensively use WhatsApp [16-18]. This app's multiple content and delivery options facilitate integrating BCTs into message design. Moreover, WhatsApp allows documentation of intervention delivery processes, including records of scheduled, sent, read, and answered messages.

As self-designed products can convey more significance to users [19] based on an increased sensation of authenticity and usefulness [20], in this study, we implement an intervention to support ART adherence through WhatsApp messages with self-designed messages in one group and standardized messages in the second group under the hypothesis of more effectiveness for the group of self-design.

For the main analysis, we will compare the efficacy of both types of messages using the Simplified Medication Adherence Questionnaire (SMAQ), a validated ART adherence questionnaire measured at 16 weeks of intervention. As the use of mHealth is incipient in our setting, the study protocol includes secondary mixed-methods components aimed at understanding the acceptability of the intervention and the challenges participants face throughout its delivery.

## Methods

### Setting

In 2023, overall, 87.1%-90% of Peruvian people between 18 and 29 years of age had access to a mobile phone, with growing trends among lower socioeconomic groups [21]. The per capita country's GDP stood at US $7789.9 [22].

This study is conducted at Hospital Nacional Cayetano Heredia in Lima, Peru’s largest city and home to over 60% of people living with HIV in Peru [23]. As part of the network of health services provided by the Peruvian Ministry of Health, this hospital serves primarily people from low-resource backgrounds and provides outpatient and inpatient HIV services to over 5000 people living with HIV. Conditioned on stocks, ART is usually dispensed monthly by HIV program nurses as walk-ins on a first-come, first-served basis at the offices of the HIV program. Pregnant people living with HIV have a much closer medical follow-up than the rest of the patient population (outside children), with monthly appointments with doctors, additional counseling services at their primary care clinics, greater priority for viral load and CD4 lymphocyte testing, and regular appointments with midwives in which adherence to ART is reinforced.

Through a longstanding alliance with Instituto de Medicina Tropical Alexander von Humboldt from Universidad Peruana Cayetano Heredia, this hospital's HIV Program continuously promotes clinical and operational research.

### Study Design

A randomized controlled trial with 2 arms and a 1:1 assignment ratio will be conducted.

### Eligibility Criteria

We assessed the eligibility criteria at the first encounter with potential participants, as follows (Textbox 1).

We excluded pregnant people since their level of care differs significantly from the rest of our potential study population, which would introduce bias regarding the effects of receiving the intervention on ART adherence.

**Inclusion and exclusion criteria.**

**Inclusion criteria**

- People living with HIV receiving HIV care at the study center
- People living with HIV aged 18-29 years
- Ownership of a mobile phone that supports WhatsApp

**Exclusion criteria**

- Pregnancy
- Impossibility to read WhatsApp messages due to illiteracy or blindness

### Study Procedures—Preimplementation Phase

First, during the study's design, we consulted with health staff to better respond to the study’s population needs without disrupting routine HIV program operations. Once the study design was completed, the coordination was intended to communicate study standard procedures and facilitate the study's implementation. Our institutional review board (IRB) approved the full study protocol in July 2023. Then, we designed the web-based study platform with Infobox Latinoamérica, a Peruvian IT company. The web-based study platform is integrated with WhatsApp business and supports the delivery of personalized and standardized messages, a Chatbot for mass-sending questionnaires and continuous chat with participants. The Infobox provides technical support during the study as required.

The principal investigator (PI) completed the web-based training on BCT-Taxonomy to design the standardized messages. He created 44 standardized messages, each corresponding to one of 14 selected BCT categories, though the final format considered elements of other behavior change theories (health belief model, social support theory, and social cognitive theory). The number of standardized messages minimized the chance of participants receiving repeated messages during the study period. Table S1 in Multimedia Appendix 1 lists all standardized messages with their corresponding BCTs in the order those messages are sent to participants.

We created the randomization sequence using the REDCap (Research Electronic Data Capture) randomization module (REDCap Consortium); sex assigned at birth, educational level, current ART intake, and previous ART abandonment were the criteria for stratified randomization.

### Study Procedures—Implementation Phase

**Figure 1:**
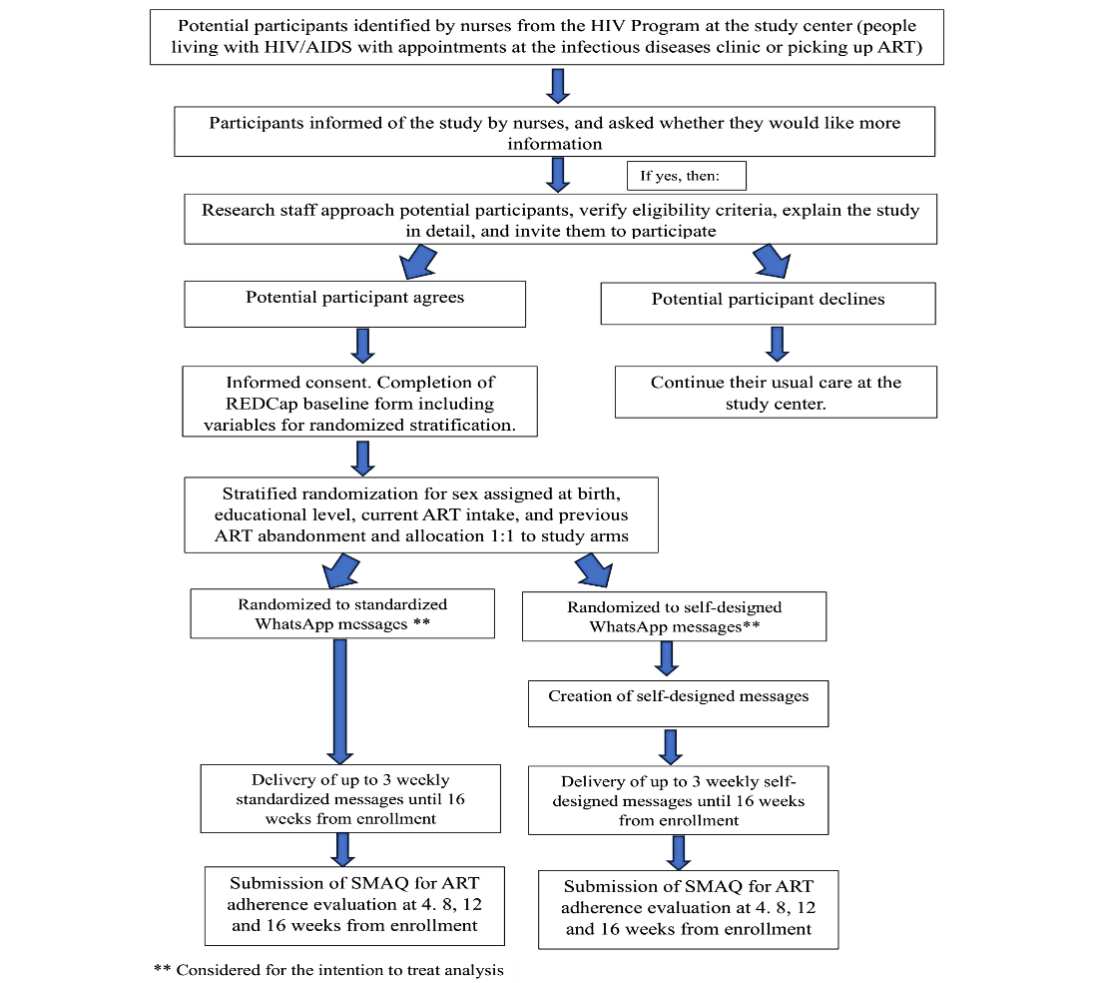
Participant identification, recruitment, and allocation. ART: antiretroviral therapy; REDCap: Research Electronic Data Capture; SMAQ: Simplified Medication Adherence Questionnaire.

To preserve confidentiality, nurses from the HIV Program are informed about the study’s eligibility criteria and collaborate to identify potential participants. While they wait for infectious diseases outpatient consults or ART pick-ups, the nurses inform them about the study. For those interested, we reassess eligibility, explain the study in detail, and obtain informed consent privately (a model of our consent form is shown in Multimedia Appendix 2). Then, we fill out a REDCap baseline questionnaire, collect data to send messages through the study web-based platform, and, when applicable, explain and support the process of self-designing the messages. Figure 1 presents participant identification, recruitment, and allocation. After completing the baseline questionnaire, which provides information for stratification (sex assigned at birth, educational level, current ART intake, and previous ART abandonment), participants are randomized to one of 2 study arms allocated with a 1:1 proportion. Then, the research staff informs participants of their assigned group and provides details accordingly. Given the characteristics of the intervention, neither the researchers nor the participants are blinded.

Participants allocated to the self-designed messages group draft 10-15 messages that would help them take their ART; this number assures similitude in the number of messages received per participant in both study arms. Message creation is done during enrollment. Participants determine the final content of their messages. However, during their creation, the research staff ensures that the content excludes potentially harmful or offensive topics. If there are any doubts during the process, the staff provides support by offering examples based on characteristics identified during the baseline questionnaires and general conversation with participants (such as family, personal hobbies, goals, and other general topics). After the messages are created, the data manager reviews them and may slightly modify them to ensure clarity while keeping the original content. Once the messages are ready, the data manager enters them into the study messaging platform.

During 16 weeks from the date of enrollment, participants receive up to 3 intervention messages per week; they are requested to reply briefly once they read those messages. Weekly, the data manager schedules the delivery of messages according to participants’ preferences, as recorded on REDCap, and monthly questionnaires. During the study, participants can chat with 2 medical doctors on the web-based study platform to ask about health issues, navigate the health system, and schedule infectious disease outpatient consultations (the standard of care requires in-person scheduling). If a participant requests modifications or additions to self-designed messages during the chat, the physician responsible for monitoring the chat will review the new messages initially. The PI will be notified if any content is deemed harmful or inappropriate. He will then contact the participant to explain the reasons for rejecting the message and will request an alternative message. This review process will continue with oversight from the data manager until the accepted messages are included in the platform’s system. Additionally, if physicians responsible for monitoring the platform identify inappropriate content during chat conversations, they will address this with all parties involved non-punitively and constructively. They will explain why the content is inappropriate and direct the conversation to ensure it remains safe and respectful.

As a follow-up, participants receive WhatsApp questionnaires every 4 weeks after enrollment and 2 days apart to assess (1) adherence to ART and (2) user experience with the intervention delivery. After 16 weeks of intervention, participants receive a final message acknowledging their participation and reminding them that the messages will stop.

Participants may withdraw from the intervention at any time by sending a message through the chat platform, responding to messages sent as part of the intervention, or contacting research team members during a medication pick-up visit. The participant will be told that withdrawing from the study does not affect their usual care at the study center. The data manager will register that patient as “withdrawn” in REDCap, and no more messages will be sent.

No concomitant care or interventions are precluded for study participants.

### Sample Size Determination

The sample size calculus considered a 95% CI and the assumption of a 10% difference in optimal ART adherence between study groups (90% in the self-designed group versus 80% in the standardized group by the end of the study). Accounting for a power level of 78.9%, the obtained result was 120 (EPIDAT 4.0). To account for a 2-week pilot phase, we increased the sample size to 131 participants.

### Outcomes

The primary outcome is ART adherence, measured by the SMAQ questionnaire at 16 weeks of intervention and adjusted for the effect of covariables. The SMAQ questionnaire evaluates medication adherence. It provides a dichotomic result of adherence (yes or no) and a semiquantitative estimate of adherence. >95% adherence is considered as “yes” and <95% as “no.”

The secondary outcomes are (1) ART adherence measured by the SMAQ Questionnaire at 4, 8, and 12 weeks of intervention; (2) loss to follow-up; (3) time of permanence in the study; and (4) metrics on the use of a bidirectional platform for intervention delivery.

Since the SMAQ provides a dichotomic result of adherence (yes or no), we will report the percentage of participants’ adherent to ART per study group at 4, 8, and 12 weeks of intervention.

To measure loss to follow-up, we defined it as either (1) participants not responding to messages for ≥30 continuous days since receiving the intervention and not responding to messages or questionnaires at the end of their intervention period (*t*=16), or (2) participants not responding to messages for ≥30 continuous days since receiving the intervention and not reading their messages until the end of their intervention period. We will measure it from the start of the delivery intervention until the completion of the study.

Time of permanence in the study will be measured from the first date of intervention delivery (baseline) until loss to follow-up or study completion (16 weeks). We will describe this result's median and interquartile range in both study arms.

To assess the use of the bidirectional platform, we will report frequencies, percentages, and means with an SD of messages and questionnaires programmed for sending, sent, received, read, and answered by participants. Also, the frequencies and percentages of different process indicators self-reported by participants.

### Data Collection

For this study, data collection and management use REDCap electronic data capture tools hosted at Universidad Peruana Cayetano Heredia [24]. The enrollment form collects participants’ baseline sociodemographic data, history of ART, strategies for taking ART, assessment of their adherence with the SMAQ, depression screening, stigma, an evaluation of their satisfaction with the health services provided at the study center, and a measurement of their knowledge about ART and adherence. For participants allocated to the self-designed messages arm, we also register their created messages in REDCap. In total, 4 members of the research team are involved in data collection. The PI trained them with mock interviews and a web-based course on using REDCap.

We used an intermediate Excel file hosted in a secured drive to schedule messaging and questionnaire delivery per participant through the web-based study platform. This file contains the identification codes, a list of messages, and the date and time of sending.

The web-based study platform collects CSV file data on every message and questionnaire scheduled, sent, received, read, and answered per participant and chat sessions with providers. The records include anonymous hour-dated logs of all conversation contents with participants, including reasons for adding, modifying, or eliminating messages in the self-designed group and responses to study questionnaires.

To obtain the total number of eligible patients who meet eligibility criteria, independent of whether they are invited to participate, we collect routine data from the study center regarding the number of outpatient consults and patients in the age group of interest who pick up ART during the study period.

### Data Management

All study data are stored in secure, password-protected platforms, with access restricted to the authors of this protocol. Members of the research staff review data collected at baseline to ensure accuracy and completeness. Weekly, we download CSV files and reports from the web-based study platform. To ensure the records' accuracy, we compare a subset with the corresponding intermediate Excel file containing messages and questionnaires scheduled for delivery. Data curation will be performed at the end of the trial to point out missing values, identify any measurement outside the expected range, and evaluate strategies for mitigating these occurrences.

### Data Analysis Plan

The primary outcome variable is ART adherence, primarily measured as a binary variable defined by the SMAQ results at 4, 8, 12, and 16 weeks of intervention. For the analysis of the primary outcome, and with an intention-to-treat approach, we will compare between both study groups the proportion of participants adherent to ART at 16 weeks of intervention with bivariate (chi-square) and multivariate (logistic regression) analyses. We will build the multivariate logistic regression model with a stepwise approach (forward selection and backward deletion), adding 1 explanatory variable at a time and starting with the explanatory variables with the highest theoretical value and strength of association with the primary outcome in bivariate analyses. At each step of model building, we will check variations in the reported odds ratios and standard errors to define the retention or removal of explanatory variables. Irrespective of bivariate association, the study group variable and potential confounders, notably history of ART abandonment, ART pick-up visits during the study period, and time of permanence in the study, will be entered into the model. We will test the final model fit with Hosmer and Lemeshow goodness-of-fit test and report results with adjusted odds ratios and 95% CIs. Preliminary outcome analyses will not be performed since they require multiple measurements, and the intervention is relatively short. We will not perform multiplicity adjustments.

With mixed models for repeated measures, we will explore the changes in ART adherence, as determined by SMAQ, from baseline to 4, 8, 12, and 16 weeks on the intention-to-treat population, with no imputation of missing data. In this model, the change from baseline value will be the dependent variable, with participant random effect and baseline ART adherence, intervention group, and measurement time as the primary fixed factors. We will check linearity assumptions for time and use participants’ unstructured covariance structure. Two-sided 95% CIs for the mean change within each study group will be calculated.

To compare the time of study permanence in both study groups, we will perform survival analysis and compare hazard ratios for study loss to follow-up between study groups, according to the study definitions. This definition lets us know how many participants are active each week of intervention.

Additionally, we will perform a descriptive analysis of participants’ baseline characteristics overall and by study groups. We will also report the percentage of participants adherent to ART per study group at 4, 8, and 12 weeks of intervention. We will describe the characteristics of the intervention delivery, including the frequencies and percentages of messages received, read, and answered by participants. We will conduct all these analyses, blinding the study groups.

We will perform a thematic analysis of the most common themes in self-designed messages to evaluate relevant qualitative outcomes. In addition, we will report the median number of messages created by participants and the main reasons for adding, modifying, or eliminating messages.

We will report the results following the CONSORT-EHEALTH (Consolidated Standards of Reporting Trials of Electronic and Mobile Health Applications and Online Telehealth) statement for randomized controlled trials [25].

### Plan for Sharing Individual Participant Data

Deidentified individual participant data collected during the trial will be made available 6 months after study completion, up to 5 years after the trial end date. Individual participant data may be shared with qualified researchers for scientific purposes. Access will be provided after a formal request, including the research proposal and statistical analysis plan, has been reviewed and approved by the principal and senior investigators, with a signed data sharing agreement. We will publish the study protocol, informed consent, and statistical analysis plan in a peer-reviewed journal. Requested data and clinical study reports will be provided through a secure data-sharing platform (Vivli—Center for Global Clinical Research Data).

### Ethical Considerations

This study protocol and all study forms, including the informed consent, have been approved by the IRBs of Universidad Peruana Cayetano Heredia (IRB00001014, Federalwide assurance number FWA00000525) and Hospital Nacional Cayetano Heredia. All amendments to this protocol are to be approved by both IRBs and amended on the trial registry’s platform. Participants sign an informed consent form before enrollment. Trained research staff give participants a verbal explanation regarding the rationale, main procedures of the study, and the main risks and benefits. They are also told that participation is voluntary, and participating, remaining, or withdrawing from the study does not affect the care they receive at the study center. Nevertheless, patients were informed that data collected until withdrawal could be used in our analysis. Messages sent by the research team do not contain allusions to the hospital, HIV, or ART, and participants are encouraged to use secure passwords on their cell phones. Participants received a 1-time compensation of US $6 to account for potential expenses in mobile data. Only the researchers can access participants’ personal data, and identifiable data are stored in secure platforms or locked cabinets. Findings will be published in peer-reviewed journals. This protocol adheres to SPIRIT (Standard Protocol Items: Recommendations for Interventional Trials) guidelines (Multimedia Appendix 3).

## Results

The study received funding from the School of Medicine of Universidad Peruana Cayetano Heredia in August 2022. The messaging platform was designed from December 2023 to March 2024. Enrollment began in March 2024, and as of July 2024, we have enrolled 131 participants. Data collection began with enrollment and intervention delivery for the first participant in March 2024. As of February 2025, we are in the process of data curation.

## Discussion

Guided by the application of BCTs, we hypothesize that self-designed messages may be more effective in improving adherence to ART than standardized messages. Therefore, we aim to evaluate the effectiveness of a mobile phone messaging intervention with WhatsApp to improve adherence to antiretrovirals in young people living with HIV who receive routine HIV care at a public health facility in Lima and assess if there is a difference in effectiveness between the 2 types of messages tested. In a setting with still inceptive use of mHealth, our study protocol will provide valuable information on associations between several covariables, ART adherence, and participants’ experience with our pilot mHealth intervention.

Self-designed messages offer customization features that may enhance participant engagement with the intervention and increase its efficacy [26,27]. Therefore, we expect participants in the self-designed messages arm to have improved adherence compared to the standardized messages arm. Some subgroups may benefit more from the intervention, although it is beyond the scope of this study to compare differences between study arms and potential effect size. Given the potential personal customization component, we hypothesize that the self-designed arm will have an increased median time of permanence in the study and lower loss to follow-up. We also anticipate that loss to follow-up may hinder the statistical significance of results for the primary outcome and ART adherence measured at 4, 8, and 12 weeks of intervention for both study arms.

A recent meta-analysis showed that SMS interventions improved adherence to ART, although there was considerable heterogeneity among study results [28]. The studies with greater efficacy were interactive or used bidirectional SMS, which supports our approach and is consistent with previous studies [11]. Our study uses a bidirectional platform, and participants can constantly interact with the research team through chatting. However, evidence suggests that implementing a messaging intervention in low-resource settings may be challenging owing to technical limitations, and difficulties in delivering the intervention may result in suboptimal outcomes [29]. Therefore, we established a 2-week pilot period that allows us to assess intervention delivery using the messaging platform, and having real-time data on multiple delivery indicators (messages and questionnaires scheduled for delivery, sent, received, seen, and answered by participants) allows for constantly monitoring the fidelity of intervention delivery.

In addition to the rigor of a randomized controlled trial, our study protocol has other strengths. To our knowledge, it is the first study evaluating the effectiveness of an mHealth intervention with different types of BCT-guided messages to improve adherence to ART. Our trial is conducted in a resource-constrained setting where there is much need for similar interventions and a still limited body of evidence about effectiveness. The thorough data register of our web-based study platform allows us to measure the cascade of intervention delivery, including the differentiation of messages received but unread.

The study also has limitations. First, as in many other studies, we use a self-reported measurement of medication adherence; extensive use does not assure full accuracy for outcome measurement. We will compare SMAQ results to viral load and CD4 lymphocyte measurements when available to address this. Limitations inherent to the local health system might prevent participants from having up-to-date results of these objective indicators, which restricts the availability of more substantial outcomes related to HIV care. Second, the sample size corresponds to a 10% difference between study groups under the assumption that participants will respond to the final adherence questionnaire, which may not be the case. Third, despite the stratified randomization, it may not be possible to identify if a particular subgroup will benefit more from the intervention. By doing a subgroup analysis of adherence, we will be able to assess the difference in outcomes among subgroups.

To conclude, this study will generate valuable evidence on the use and implementation of mHealth services for young people living with HIV in Peru. If effective, it could inform public policies and improve the quality of care for young people living with HIV.

## Appendix

Multimedia Appendix 1 Standardized messages and their associated behavioral change technique.

Multimedia Appendix 2 Informed consent (original, Spanish).

Multimedia Appendix 3 SPIRIT Checklist.

## Abbreviations

ART: antiretroviral therapy
BCT: behavioral change technique
CONSORT-EHEALTH: Consolidated Standards of Reporting Trials of Electronic and Mobile Health Applications and Online Telehealth
IRB: institutional review board
mHealth: mobile health
PI: principal investigator
REDCap: Research Electronic Data Capture
SMAQ: Simplified Medication Adherence Questionnaire
SPIRIT: Standard Protocol Items: Recommendations for Interventional Trials
