# Supplementary material for: Intervention With WhatsApp Messaging to Compare the Effect of Self-Designed Messages and Standardized Messages in Adherence to Antiretroviral Treatment in Young People Living With HIV in a Hospital in Lima, Peru: Protocol for a Nonblinded Randomized Controlled Trial
Source: JMIR Res Protoc. 2025 May 22;14:e66941. doi: 10.2196/66941 (PMC12141961; doi:10.2196/66941)
Supplement: Multimedia Appendix 1 [file resprot_v14i1e66941_app1.docx]

Table S1. Standardized messages and their associated Behavioral Change Technique (BCT)

| **Original message (Spanish)** | **Message (English)** | **Associated BCT** |
| --- | --- | --- |
| Hola^a^, estar informados siempre es mejor. En caso tengas dudas, consulta con nosotros a este número. Estamos aquí para apoyarte. | Hello, being informed is always better. If you have any questions, contact us at this number. We are here to support you. | 3.1 Social support (unspecified) |
| Hola, piensa qué dificultades has tenido en el pasado para tomar tu medicación y asistir a tus citas. Considera cómo puedes superarlas. ¡Tú tienes el poder de hacerlo! | Hello, think about the difficulties you’ve had in the past with taking your medication and attending your appointments. Consider how you can overcome them. You have the power to do it! | 1.2 Problem solving |
| Hola, la medicación se debe tomar todos los días en horarios regulares, aunque te sientas bien. | Hello, medication should be taken every day at regular times, even if you feel well. | 4.1 Instruction on how to perform the behavior. |
| Hola, si no puedes asistir a tu cita, reprográmala escribiendo a este número. ¡No la pierdas! Te queremos saludable. | Hello, if you cannot attend to your appointment, reschedule it by writing to this number. Don’t miss it! We want you to stay healthy. | 3.1 Social support (unspecified) |
| Hola, tomar tu medicación como debes te ayudará a mantenerte sano. Queremos lo mejor para ti. ¡Tú puedes! | Hello, taking your medication as prescribed will help you stay healthy. We want the best for you. You can do it! | 5.1 Information about health consequences |
| Hola, si no tomas tu medicación siempre, incluso cuando te sientes sano y bien, pones en riesgo tu salud. | Hello, if you don’t always take your medication, even when you feel healthy and well, you put your health at risk. | 5.1 Information about health consequences |
| Hola, si cuidas de tu salud siempre como debes, considera darte un premio. ¡Te lo mereces! | Hello, if you always take care of your health as you should, consider rewarding yourself. You deserve it! | 10.9 Self-reward |
| Hola, muchos no acuden a sus citas cuando deben, pero confiamos en que tú sí lo harás. Sé parte del cambio, ayúdanos a mejorar. | Hello, many people miss their appointments, but we trust that you won’t. Be part of the change, help us improve. | 6.2 Social comparison |
| Hola, cuidar de tu salud y hacer lo correcto te ayudará a ganar confianza en lograr todo lo que te propones. | Hello, taking care of your health and doing the right thing will help you gain confidence in achieving everything you set your mind to. | 5.6 Information about emotional consequences |
| Hola, si sientes molestias al tomar tu medicación (náuseas, vómitos, diarrea; etc.), escríbenos a este número. Puedes contar con nosotros. | Hello, if you experience discomfort when taking your medication (nausea, vomiting, diarrhea, etc.), write to us at this number. You can count on us. | 3.1 Social support (unspecified) |
| Hola, puedes probar tomar tu medicación todos los días luego de lavarte los dientes. Así, se te hará más fácil acordarte. | Hello, you can try taking your medication every day after brushing your teeth. That way, it will be easier to remember it. | 8.3 Habit formation |
| Hola, si prefieres, puedes poner un recordatorio en el celular o en lugares visibles para ayudarte a hacer lo que debes hacer. | Hello, if you prefer, you can set a reminder on your phone or place notes in visible areas to help you do what you need to do. | 7.1 Prompts/cues |
| Hola, piensa cómo puedes acordarte mejor de tomar tu medicación. Estamos aquí para lo que necesites. | Hello, think about how you can better remember to take your medication. We are here for whatever you need. | 1.2 Problem solving |
| Hola, considera qué te impide tomar tu medicación como deberías, y toma acción para solucionarlo. Estamos en esto juntos! | Hello, consider what prevents you from taking your medication as you should, and take action to solve it. We are in this together! | 1.2 Problem solving |
| Hola, tomar tu medicación como lo indicado es importante para prevenir consecuencias graves. Si tienes alguna duda, puedes escribirnos. | Hello, taking your medication as prescribed is important to prevent serious consequences. If you have any doubts, feel free to write to us. | 5.1 Information about health consequences |
| Hola, rodéate de personas que solo quieren lo mejor para ti y te ayudan a cuidarte. | Hello, surround yourself with people who only want the best for you and help you take care of yourself. | 12.2 Restructuring the social environment |
| Hola, cuidar de tu salud y acudir a tus citas te hará sentir bien y satisfecho. | Hello, taking care of your health and attending your appointments will make you feel good and satisfied. | 5.6 Information about emotional consequences |
| Hola, piensa en cómo te sientes cuando no tomas tu medicación como debes. Estamos contigo. | Hello, think about how you feel when you don’t take your medication as you should. We are here for you. | 5.5 Anticipated regret |
| Hola, ten a la mano tu medicación para que recuerdes tomarla cuando debes. | Hello, keep your medication at hand so you remember to take it when scheduled. | 7.1 Prompts/cues |
| Hola, los expertos recomiendan cuidar de la salud y tomar la medicación todos los días para ayudar a mantenerte sano. Así te queremos siempre. | Hello, experts recommend taking care of your health and taking your medication every day to help you stay healthy. That’s how we always want to see you. | 9.1 Credible source |
| Hola, la medicación solo funciona cuando la tomas todos los días como lo indicado. Tú puedes!! | Hello, medication only works when you take it every day as prescribed. You can do it! | 5.1 Information about health consequences |
| Hola, asistir a tus citas te permitirá saber cómo estás de salud y resolver tus dudas. Cualquier cosa, escríbenos. Queremos que estés bien! | Hello, attending your appointments will allow you to check on your health and get your questions answered. If you need anything, please reach out to us. We want you to be well! | 5.1 Information about health consequences |
| Hola, si prefieres, puedes tener lo que tienes que tomar a la mano, o en un lugar visible para que recuerdes tomarlas. | Hello, if you prefer, keep what you need to take within reach or in a visible place, so you remember to take it. | 12.1 Restructuring the physical environment |
| Hola, tomar tu medicación como lo indicado te hará sentir más satisfecho, y mejor contigo mismo. | Hello, taking your medication as prescribed will make you feel more satisfied and better about yourself. | 5.6 Information about emotional consequences |
| Hola, recuerda pasar más tiempo con las personas que te apoyan y te ayudan a mantenerte saludable. Queremos verte bien!! | Hello, remember to spend more time with people who support you and help you stay healthy. We want to see you well! | 12.2 Restructuring the social environment |
| Hola, si te sientes abrumado y te es difícil recordar tomar tu medicación, respira hondo, haz una lista de los problemas que tienes y qué acciones puedes tomar para resolverlos. El cambio depende de ti! | Hello, if you feel overwhelmed and find it hard to remember to take your medication, take a deep breath, make a list of the problems you have, and consider actions you can take to solve them. The change is in your hands! | 11.2 Reduce negative emotions |
| Hola, piensa en hábitos saludables que has logrado tener. Así debes hacer para cuidar tu salud!! | Hello, think about the healthy habits you’ve already managed to develop. That’s how you should take care of your health! | 15.3 Focus on past success |
| Hola, menos de la mitad de personas toma su medicación todos los días como debería. Sé parte del cambio. Ayúdanos a mejorar. | Hello, less than half of people take their medication every day as they should. Be part of the change. Help us improve. | 6.2 Social comparison |
| Hola, los expertos recomiendan buscar ayuda emocional y consejería si te sientes abrumado. Recuerda, estamos aquí para cuidarte. | Hello, experts recommend seeking emotional support and counseling if you feel overwhelmed. Remember, we are here to take care of you. | 9.1 Credible source |
| Hola, si acudes a tus citas siempre como debes, considera darte un premio. ¡Te lo mereces! | Hello, if you always attend your appointments as you should, consider rewarding yourself. You deserve it! | 10.9 Self-reward |
| Hola, tú tienes el poder de hacer el cambio y cuidar de tu salud. ¡Creemos en ti! | Hello, you have the power to make the change and take care of your health. We believe in you! | 15.1 Verbal persuasion about capability |
| Hola, imagina cómo sería tomar tu medicación siempre y sentirte bien sabiendo que te mantienes saludable. | Hello, imagine what it would be like to always take your medication and feel good knowing you are staying healthy. | 16.2 Imaginary reward |
| Hola, considera pasar menos tiempo con personas que te impiden tomar tu medicación, asistir a tus citas y cuidar de tu salud. Queremos lo mejor para ti! | Hello, consider spending less time with people who prevent you from taking your medication, attending your appointments, and taking care of your health. We want the best for you! | 12.2 Restructuring the social environment |
| Hola, si cuidas de tu salud, tomas tu medicación y asistes a tus citas, serás un buen ejemplo para las personas que te rodean. | Hello, if you take care of your health, take your medication, and attend your appointments, you will be a great example for those around you. | 13.1 Identification of self as role model |
| Hola, tú tienes el poder de tomar tu medicación, asistir a tus citas y mantenerte saludable. ¡Adelante! | Hello, you have the power to take your medication, attend your appointments, and stay healthy. Keep going! | 15.1 Verbal persuasion about capability |
| Hola, piensa en hábitos saludables que ya has logrado tener. Si pudiste antes, también puedes ahora. Así debes hacer para tomar tu medicación y cuidar de tu salud. | Hello, think about the healthy habits you have already managed to develop. If you could do it before, you can do it again. That’s how you should take your medication and take care of your health. | 15.3 Focus on past success |
| Hola, tomar tu medicación todos los días y mantenerte saludable está en tus manos. ¡Creemos en ti! | Hello, taking your medication every day and staying healthy is in your hands. We believe in you! | 15.1 Verbal persuasion about capability |
| Hola, cuando tomes tu medicación, recuerda por qué lo haces, y piensa que lo tienes que hacer siempre. Hay que estar convencidos de hacer lo correcto. | Hello, when you take your medication, remember why you do it and think about how you need to do it always. You have to be convinced to do the right thing. | 15.4 Self talk |
| Hola, imagina tomar tu medicación y estar saludable y feliz. Así te queremos ver siempre!! | Hello, imagine taking your medication and being healthy and happy. That’s how we always want to see you! | 15.2 Mental rehearsal of successful performance |
| Hola, piensa en las veces que sí te acordaste de tomar tu medicación, y recuerda que debe volverse un hábito. | Hello, think about the times you did remember to take your medication, and remember that it should become a habit. | 15.3 Focus on past success |
| Hola, cuando acudes a tus citas, piensa que es por tu bien. Estamos para ayudarte. | Hello, when you attend your appointments, remember that it’s for your own good. We are here to help you. | 15.4 Self talk |
| Hola, imagina todas las cosas que puedes lograr si tomas tu medicación y te mantienes saludable. Así te queremos!! | Hello, imagine all the things you can achieve if you take your medication and stay healthy. That’s how we want you to be! | 16.2 Imaginary reward |
| Hola, piensa que tomar tu medicación te ayudará a mantenerte mejor para ti y quienes te quieren! Tú puedes lograrlo :) | Hello, think about how taking your medication will help you stay well for yourself and those who love you! You can do it :) | 15.4 Self talk |
| Hola, piensa que cuidando de tu salud podrás disfrutar con los que más quieres, y eso es lo mejor. Toma tu medicación, asiste a tus citas. Te queremos saludable siempre!! | Hello, think about how taking care of your health will allow you to enjoy time with the ones you love the most, and that’s the best thing. Take your medication, attend your appointments. We want you to stay healthy always!! | 16.2 Imaginary reward |

^a^ Our web platform allowed coded programming of the participant's name so that each standardized message begins with a greeting, such as “Hello [name]”.
