# Supplementary material for: Intervention With WhatsApp Messaging to Compare the Effect of Self-Designed Messages and Standardized Messages in Adherence to Antiretroviral Treatment in Young People Living With HIV in a Hospital in Lima, Peru: Protocol for a Nonblinded Randomized Controlled Trial
Source: JMIR Res Protoc. 2025 May 22;14:e66941. doi: 10.2196/66941 (PMC12141961; doi:10.2196/66941)
Supplement: Multimedia Appendix 2 [file resprot_v14i1e66941_app2.docx]

Informed consent (original, Spanish)

| CONSENTIMIENTO INFORMADO PARA PARTICIPAR EN UN ESTUDIO DE INVESTIGACIÓN | |
| --- | --- |
| (Adultos) | |
| Título del estudio: | Intervención con mensajería por WhatsApp para evaluar el efecto de mensajes autodiseñados y estandarizados en la adherencia al tratamiento antirretroviral en PVVS jóvenes de un hospital en Lima-Perú: ensayo clínico aleatorizado |
| Investigador principal: | Jeffrey Nathan Freidenson Bejar |
| Institución: | Universidad Peruana Cayetano Heredia |

Propósito del estudio:

Lo estamos invitando a participar en un estudio para evaluar la utilidad del uso de mensajes de WhatsApp para la correcta toma de los medicamentos antirretrovirales en personas que toman el Tratamiento Antirretroviral (TARV). Este es un estudio desarrollado por investigadores de la Universidad Peruana Cayetano Heredia (UPCH), en colaboración con la Estrategia Sanitaria Nacional de Control de VIH/SIDA del Hospital Cayetano Heredia (HCH).

El Virus de la Inmunodeficiencia Humana (VIH) se puede controlar gracias al TARV. Sin embargo, es necesario que esa medicación sea tomada tal y como su médico lo prescribe, para no perder los beneficios del TARV.

Los mensajes de carácter motivacional a través de celulares para apoyar a pacientes VIH con la correcta toma de TARV se ha usado en diferentes países con resultados muy positivos. En este estudio, queremos seguir explorando qué características de los mensajes hacen que funcionen mejor para los pacientes.

Información sobre la intervención y los mensajes de texto:

Si decide participar en este estudio, se procederá con lo siguiente:

1. Se registrará el número celular de uso personal al cual se enviarán los mensajes deWhatsApp.
2. Si accede a ello, se recolectará información archivada en su historia clínica relacionada al VIH (fecha de diagnóstico, fecha de inicio de tratamiento, marcadores de CD4 y carga viral, cuestionario SMAQ anteriores) con el propósito de conocer aspectos de su condición.
3. Será asignado de manera aleatoria, al azar, a una de las dos formas de mensajes consideradas en este estudio.

Grupo A: Mensajes elaborados por el equipo de investigación (“estandarizados").

Grupo B: Mensajes elaborados por el participante (“autodiseñados”).

* Los participantes asignados al grupo B pasarán con uno de los miembros del equipo de investigación, quien le dará algunas pautas para la creación de mensajes. Al finalizar con dicha creación, se le preguntará sobre su satisfacción durante el proceso.

Ambos grupos compartirán las siguientes características

1. Mensajes bidireccionales: Usted también podrá mandar mensajes a nuestro equipo con alguna inquietud o pregunta relacionada a su atención en el hospital, usando el celular en el que recibe los mensajes del estudio. El equipo encargado procurará responderle en 24 horas, de acuerdo a disponibilidad. Si la respuesta del equipo demora >24 horas, se le contactará en el menor tiempo posible.

2. Los mensajes serán enviados con una frecuencia de 1-3 veces por semana, siendo 1 el mínimo y 3 el máximo. Por favor, responda a los mensajes del estudio para asegurarnos que hayan sido recibidos. Anotaremos sus preferencias con respecto al horario y día de la semana en el que desee recibir los mensajes. Por otro lado, usted podrá cambiar los días y horas de envío esccribiendo al equipo mediante el chat (WhatsApp), al número que envía los mensajes.Los participantes asignados al grupo B tendrán la oportunidad de cambiar sus mensajes comunicando esto al equipo mediante el chat mencionado. El envío de mensajes está planeado por 4 meses a partir del envío del primer mensaje.

3. Durante el periodo de intervención de 4 meses, se le enviará 2 cuestionarios breves mediante WhatsApp para conocer algunos aspectos de la toma de medicación (cuestionario SMAQ de adherencia), y aspectos del envío de mensajes (cuestionario de indicadores de proceso). Estos le serán enviados 1 vez por mes desde que inició su participación en el estudio (al cumplir el mes 1, 2, 3 y 4), condicionado a que continúe su participación en el estudio. Estos cuestionarios breves toman aproximadamente 2-3 minutos en conjunto (para los 2, si los hace seguidos), y se completarán en WhatsApp a manera de “chatbot” (marcando respuestas a las preguntas que se le envíen en el chat).

4. Tres meses después de haber recibido el último mensaje del estudio, lo volveremos a contactar para preguntarle sobre su toma de medicación y conocer los potenciales efectos de nuestra intervención en dicho momento.

Riesgos:

Al participar en este estudio, hay un riesgo potencial si alguien no autorizado revisa su celular y lee los mensajes de este estudio. Para reducir ese riesgo, procure:

1. Cuidar dónde y frente a quiénes emplea su celular.

2. Proteger el uso de su celular con una contraseña privada.

3. Eliminar los mensajes después de leerlos.

Para quienes reciban mensajes elaborados por los investigadores, (Grupo A), podemos asegurar que estos son de carácter motivacional y no contienen ninguna alusión ni mención a vivir con VIH. El único término parcialmente relacionado es la palabra “medicación”, pero nada explícito relacionado al VIH. Si usted participa del grupo B, decidirá el contenido de sus mensajes, con ayuda de un miembro de nuestro equipo, si fuera necesario. El contenido de los mensajes autodiseñados podría ser ligeramente modificado por nuestro equipo para poder ser aprobado por la plataforma que brinda los servicios de mensajería.

Beneficios:

Algunos estudios previos sobre el uso de los mensajes por medio de celulares para mejorar el cumplimiento con la toma del TARV han tenido buenos resultados. Por lo tanto, usted podría verse beneficiado por estos efectos al recibirlos.

Costos y compensación

No deberá pagar nada por participar en el estudio. En reconocimiento a posibles gastos derivados de su participación, usted recibirá S/. 20 (veinte y 0/00 soles) para reponer para reponer consumos de datos de celular ocasionados por el uso de la mensajería, o como reembolso de una consulta médica en el centro de estudio.

Confidencialidad:

Nosotros guardaremos su información con códigos y no con nombres. Si los resultados de este seguimiento son publicados, no se mostrará ninguna información que revele su identidad.

USO FUTURO DE INFORMACIÓN

Deseamos conservar la información recolectada por 10 años. La información solo se identificará con códigos, su nombre no aparecerá en ningún momento ni habrá forma de ubicarlo.

Si no desea que la información sea almacenada ni utilizada posteriormente, aún puede seguir participando del estudio. En ese caso, terminada la investigación sus datos serán eliminados.

La información de sus resultados será guardada y usada posteriormente para estudios de investigación beneficiando al mejor conocimiento del uso de estas tecnologías, permitiendo la evaluación de medidas de control de la toma del TARV para su implementación en el centro de estudio

Previamente al uso de sus datos en un futuro proyecto de investigación, ese proyecto contará con el permiso de un Comité Institucional de Ética en Investigación.

Autorizo a tener mis datos almacenados por 10 años para un uso futuro en otras investigaciones. (Después de este periodo se eliminarán).

SI ( ) NO ( )

Derechos del participante:

Si decide participar en el estudio, puede retirarse de éste en cualquier momento, o no participar en una parte del estudio sin daño alguno. Si tiene alguna duda adicional, por favor pregunte al personal del estudio o comuníquese con el Investigador principal/Coordinador del estudio: Jeffrey Freidenson, al correo electrónico jeffrey.freidenson.b@upch.pe

Si tiene preguntas sobre los aspectos éticos del estudio, o cree que ha sido tratado injustamente puede contactar al Dr. Manuel Raúl Pérez Martinot, presidente del Comité Institucional de Ética en Investigación de la Universidad Peruana Cayetano Heredia al teléfono 01-3190000 anexo 201355 o al correo electrónico: [orvei.ciei@oficinas-upch.pe](mailto:duict.cieh@oficinas-upch.pe)

Asimismo, puede ingresar a este enlace para comunicarse con el Comité Institucional de Ética en Investigación UPCH: <https://investigacion.cayetano.edu.pe/etica/ciei/consultasoquejas>

Una copia de este consentimiento informado le será entregada.

}

DECLARACIÓN Y/O CONSENTIMIENTO

Acepto voluntariamente participar en este estudio y comprendo las actividades en las que participaré si decido hacerlo. También entiendo que puedo decidir no participar y que puedo retirarme del estudio en cualquier momento.

| Nombres y Apellidos Participante |  | Firma |  | Fecha y Hora |
| --- | --- | --- | --- | --- |
| Nombres y Apellidos  Testigo (si el participante es analfabeto |  | Firma |  | Fecha y Hora |
| Nombres y Apellidos Investigador |  | Firma |  | Fecha y Hora |
